# O10.3. EARLY BRAIN AND COGNITIVE DEVELOPMENT IN CHILDREN AT RISK FOR SCHIZOPHRENIA

**DOI:** 10.1093/schbul/sby015.255

**Published:** 2018-04-01

**Authors:** Veronica Murphy, Sarah Short, Emil Cornea, Barbara Goldman, Gang Li, Dinggang Shen, John Gilmore

**Affiliations:** 1 University of North Carolina; 2 University of Wisconsin

## Abstract

**Background:**

Currently, most attempts at early identification and intervention for individuals at risk for schizophrenia focus on the prodromal phase of the illness during adolescence. However, cognitive and other deficits likely arise well before the prodromal phase. Many risk genes for schizophrenia play a role in early brain development, and recent studies indicate that the basic structural and functional networks of the brain are in place by the second year of life. This suggests that schizophrenia likely has origins in prenatal and early childhood brain development, and that early identification and intervention may need to be shifted to this developmental period to have a real impact on the incidence and severity of schizophrenia.

**Methods:**

We studied early childhood brain development 25 children of mothers with schizophrenia and 178 control children. Children had a 3T MRI after birth and at 1 and 2 years of age, and global tissue volumes (gray matter, white matter, CSF), ventricle volumes, and cortical thickness and surface area were determined. Children were also assessed with the Mullen Scales of Early Learning at 1 and 2 years.

**Results:**

Children at risk for schizophrenia had significantly lower Mullen Composite scores at both age 1 (p=0.0078) and 2 years (p=0.0001) compared to control children. Reductions were present in fine motor, expressive and receptive language scales at both ages. Overall, high-risk children did not differ from controls in global tissue volumes, though there was evidence of a gender effect. Female high-risk children tended to have reduced gray matter volumes after birth and at age 1 year (significant reduction after birth, p =0.018), while males tended have increased gray matter volumes at age 1 and 2 years (significant at 1 year, p = 0.037). Cortical thickness and surface area results tended to reflect the gray matter volume findings. Females had regions of significant cortical surface area reduction after birth, while males had several regions of significant of cortical surface area expansion at 1 year. Males had a few regions of significant changes of cortical thickness after birth.

**Discussion:**

In the context of its limitations, this study confirms previous studies that find alterations of very early childhood development in children at risk for schizophrenia. It also indicates that alterations of cortical gray matter are evident in very early childhood, and that there is a gender difference in these alterations, with females having reduced gray matter volumes and males having increased gray matter volumes. Brain structure and cognitive abnormalities associated with risk for schizophrenia are present shortly after birth; future studies may be able to identify very early biomarkers of risk that will not only improve our understanding of how brain abnormalities associated with schizophrenia develop, but also define periods of childhood development that can be targeted with early intervention.

